# Ingestion of Leucine + Phenylalanine with Glucose Produces an Additive Effect on Serum Insulin but Less than Additive Effect on Plasma Glucose

**DOI:** 10.1155/2013/964637

**Published:** 2013-07-29

**Authors:** Jennifer F. Iverson, Mary C. Gannon, Frank Q. Nuttall

**Affiliations:** ^1^Endocrinology, Metabolism, and Nutrition Section, Minneapolis VA Health Care System, One Veterans Drive, Minneapolis, MN 55417, USA; ^2^Department of Medicine, University of Minnesota, Minneapolis, MN 55455, USA; ^3^Department of Food Science and Nutrition, University of Minnesota, St. Paul, MN 55108, USA

## Abstract

Most individual amino acids stimulate insulin secretion and attenuate the plasma glucose response when ingested with glucose. We determined whether ingestion of two amino acids simultaneously with glucose would result in an additive effect on the glucose area response compared with ingestion of amino acids individually. Leucine and phenylalanine were chosen because they were two of the most potent glucose-lowering amino acids when given individually. Eight healthy subjects were studied on four separate days. Test meals were given at 0800. The first meal was a water control. Subjects then received 25 g glucose or leucine + phenylalanine (1 mmol/kg fat free body mass each) ±25 g glucose in random order. Glucose, insulin and glucagon were measured frequently for 2.5 hours thereafter. Net areas under the curves were calculated using the mean fasting value as baseline. The insulin response to leucine + phenylalanine was additive. In contrast, the decrease in glucose response to leucine + phenylalanine + glucose was less than additive compared to the individual amino acids ingested with glucose. Interestingly, the insulin response to the combination was largely due to the leucine component, whereas the glucose response was largely due to the phenylalanine component. Glucose was unchanged when leucine or phenylalanine, alone or in combination, was ingested without glucose. This trial is registered with ClinicalTrials.gov NCT01471509.

## 1. Introduction

In a series of studies, our laboratory has quantified the ability of single amino acids, when ingested at 1.0 mmol/kg fat-free body mass with or without 25 g of glucose, to stimulate a rise in insulin and/or glucagon. Also, when ingested with glucose, their potential to attenuate the glucose area response integrated over a 2.5-hour period of time was determined. Sixteen amino acids were studied. The potency of the individual amino acids in regard to their effect on the insulin, glucagon, and glucose concentrations varied greatly between amino acids, and the specific responses could not be predicted based on the structure of the amino acids [1].

Leucine and phenylalanine were particularly potent in their ability to attenuate the glucose response to 25 g of glucose. Therefore, we were interested in determining if the glucose-attenuating effect would be greater if these two amino acids were ingested together with glucose. We were particularly interested in whether the effects would be additive. The effects of these amino acids on the insulin and glucagon responses also were monitored.

## 2. Methods

Eight healthy subjects (four men and four women) were studied. The mean age of the subjects was 26.5 years (range 22–32). Their mean weight was 71.1 kg (range 50.0–89.6) with a mean fat-free mass of 53.2 kg (range 36.4–68.9) and a mean body mass index of 23.3 (range 18.9–27.5).

Written informed consent was obtained from all subjects. The study was approved by the Minneapolis Department of Veterans Affairs Medical Center and the University of Minnesota Committees on Human Subjects and filed with ClinicalTrials.gov (NCT01471509). Fat-free body mass was determined using a bioelectrical impedance analyzer (RJL Systems Inc., Clinton Township, MI, USA). Baseline laboratory studies including thyroid, liver, and kidney function as well as fasting plasma glucose, hemoglobin A1c, and lipid profiles were within the normal reference ranges.

Each subject was admitted to the Special Diagnostic and Treatment Unit (SDTU) on four separate mornings. Subjects were asked to fast for 12 hours prior to testing. On the first day subjects were given 120 mL water only as a fasting control. At subsequent visits, the following three test meals were given in random order: (1) 25 g glucose (45 mL Glutol), (2) L-leucine + L-phenylalanine (1 mmol/kg fat-free body mass each), and (3) 25 g glucose with L-leucine + L-phenylalanine (1 mmol/kg fat-free body mass) each with 120 mL water. All amino acids were kindly provided by Ajinomoto, Inc. (Raleigh, NC, USA). The amino acid dose of 1 mmol/kg fat-free body mass was chosen because it is approximately the amount of an amino acid that would be consumed during a high-protein meal. An indwelling catheter was inserted into an antecubital vein and kept patent with intravenous saline. Baseline blood samples were drawn at 7:40, 7:50, and 8:00 AM. Then, blood samples were obtained every ten minutes for 120 minutes and at 150 minutes after ingestion of each test meal for determination of plasma glucose, serum insulin, and plasma glucagon concentrations. 

Net areas under the curve for each parameter were calculated using a computer program based on the trapezoid rule with the mean fasting value used as baseline. Data for each amino acid pair was then compared to data we had previously obtained for each of the component individual amino acids [2, 3]. Ideally, we wanted to study each subject for an additional four days to allow comparisons between the individual amino acids and the amino acid pairs in the same subjects, rather than comparing the data with historical data. However, given the longer time commitment required and difficulty in recruiting subjects for an eight-day study, this design was not practical.

Plasma glucose concentrations were determined by a hexokinase method using an Abbott Architect ci8200 analyzer (Abbott Laboratories, Abbott Park, IL, USA). Serum immunoreactive insulin was measured using an automated chemiluminescent assay on Siemens IMMULITE analyzer. Plasma glucagon was determined by radioimmunoassay using kits purchased from Millipore (Billerica, MA, USA).

Statistics were determined using Student's *t* test for paired variates with the StatView 512+ program (Abacus Concepts, Calabasas, CA, USA) for the Macintosh computer (Apple, Cupertino, CA, USA). A *P* value less than 0.05 was the criterion used for significance. Data are presented as means (±SEMs).

## 3. Results

**Figure 1:**
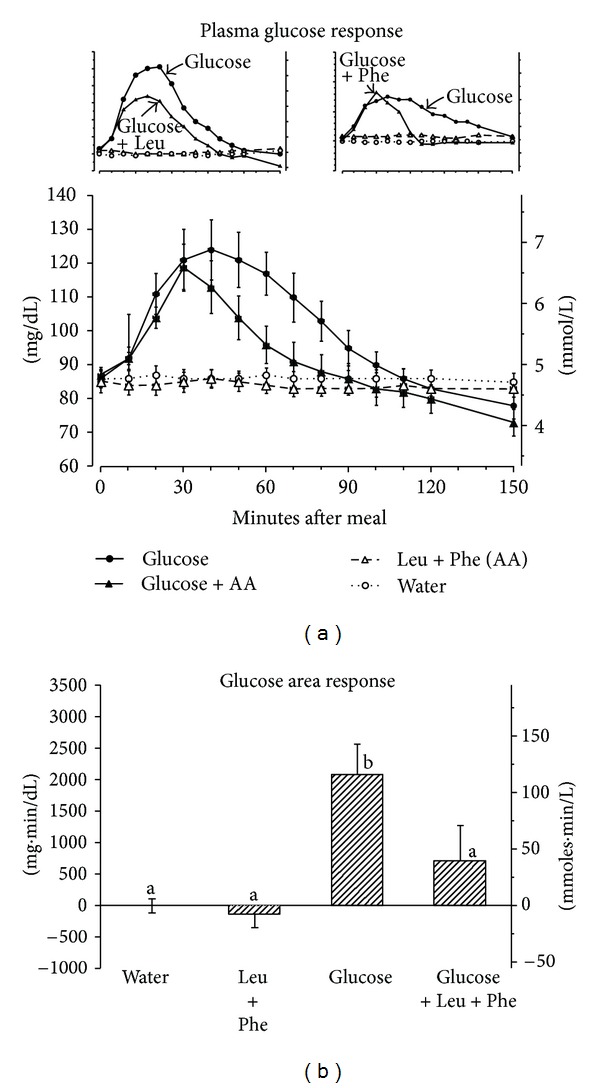
(a) Mean (±SEM) plasma glucose concentration in 8 healthy subjects after ingestion of water only (open circles), 25 g glucose (closed circles), leucine + phenylalanine at 1 mmol/kg fat-free mass each (open triangles), or 25 g glucose with leucine + phenylalanine at 1 mmol/kg fat-free mass each (closed triangles). Insert left—previous data with leucine alone [2]. Insert right—previous data with phenylalanine alone [3]. (b) Net-integrated AUC using the fasting values as baseline. Bars with different letters indicate that values are significantly different (*P* < 0.05).

The mean fasting glucose concentration was 86 mg/dL (Figure 1(a)). After ingestion of glucose alone the mean plasma glucose concentration reached a peak of 124 mg/dL at 40 minutes and then slowly returned to the fasting concentration by 110 minutes. In contrast, when the subjects ingested glucose with leucine + phenylalanine the mean peak glucose concentration was lower (119 mg/dL) and was reached more quickly (30 minutes versus 40 minutes post-ingestion) and returned to baseline more quickly (90 minutes versus 110 minutes). However, the glucose was continuing to decrease at the end of the study in both cases. Ingestion of leucine + phenylalanine without glucose had only a very modest effect on the plasma glucose level.

The glucose area response (Figure 1(b)) decreased by 66% when leucine + phenylalanine were ingested with glucose compared to ingestion of glucose alone (*P* = 0.027). The glucose response to leucine + phenylalanine alone was slightly less than that for water alone, but this did not reach statistical significance.

**Figure 2:**
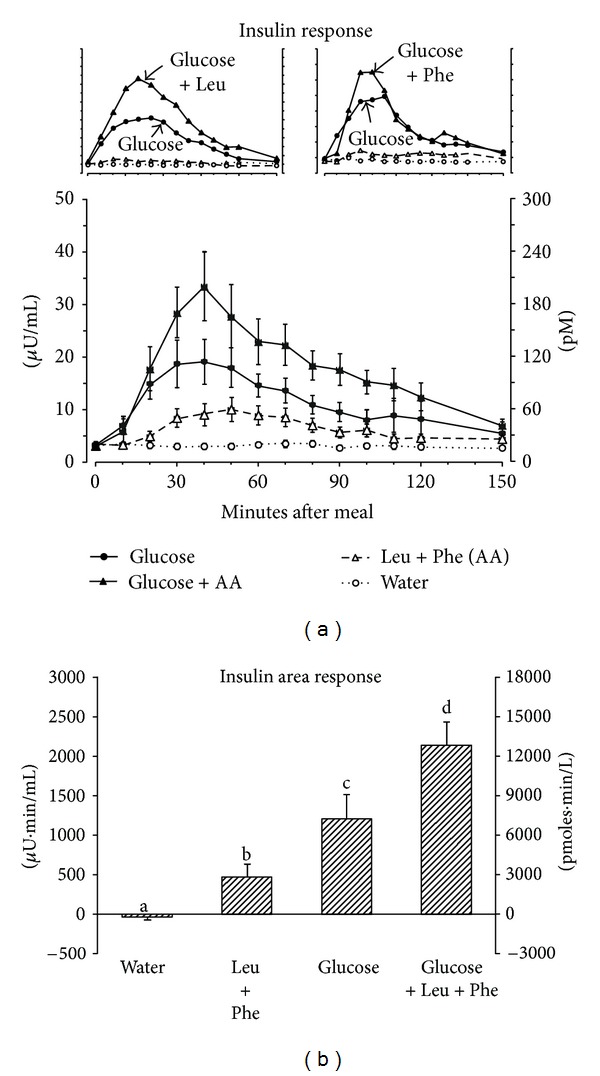
(a) Mean (±SEM) serum insulin concentration in 8 healthy subjects after ingestion of water only (open circles), 25 g glucose (closed circles), leucine + phenylalanine at 1 mmol/kg fat-free mass each (open triangles), or 25 g glucose with leucine + phenylalanine at 1 mmol/kg fat-free mass each (closed triangles). Insert left—previous data with leucine alone [2]. Insert right—previous data with phenylalanine alone [3]. (b) Net-integrated AUC using the fasting values as baseline. Bars with different letters indicate that values are significantly different (*P* < 0.05).

The mean fasting serum insulin concentration was 3.2 *μ*U/mL (Figure 2(a)). After ingestion of glucose alone the mean serum insulin concentration reached a peak of 19 *μ*U/mL at 40 minutes then gradually decreased and was very close to the baseline level by 150 minutes. When leucine + phenylalanine was ingested with glucose the mean serum insulin concentration reached a maximum that was 75% higher than with glucose alone (33 *μ*U/mL); the maximum occurred at the same time as when glucose was ingested alone (40 minutes). The serum insulin concentration then declined more rapidly and thus reached the baseline at the same time as when glucose was ingested alone. Ingestion of leucine + phenylalanine without glucose resulted in a modest increase in serum insulin concentration with a maximum of 10.0 *μ*U/mL at 50 minutes post-ingestion.

The insulin area response (Figure 2(b)) was 77% greater when leucine + phenylalanine was ingested with glucose compared to ingestion of glucose alone (*P* = 0.005). Ingestion of leucine + phenylalanine without glucose resulted in a small but significantly greater insulin area response compared to water ingestion alone (*P* = 0.012).

**Figure 3:**
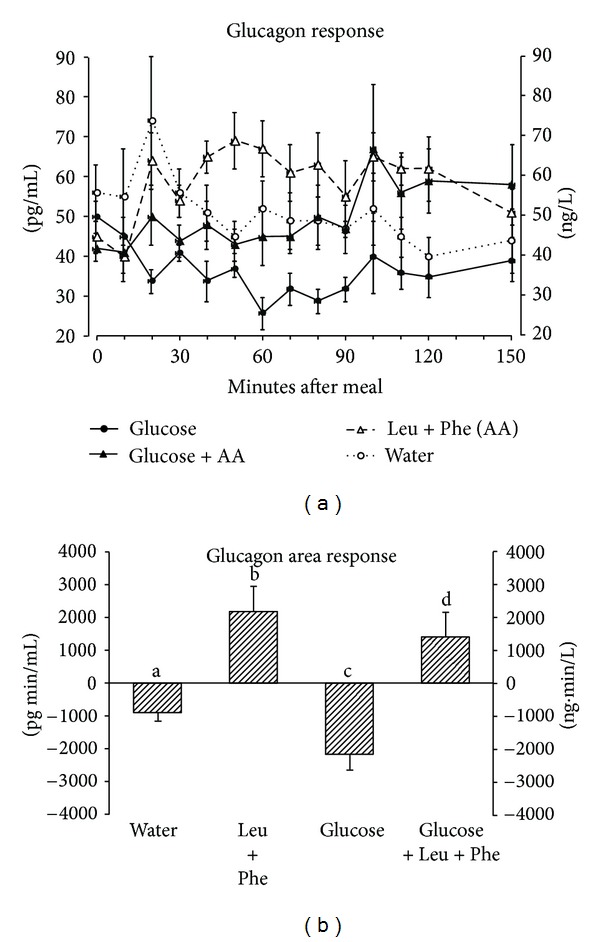
(a) Mean (±SEM) plasma glucagon concentration in 6 healthy subjects after ingestion of water only (open circles), 25 g glucose (closed circles), leucine + phenylalanine at 1 mmol/kg fat-free mass each (open triangles), or 25 g glucose with leucine + phenylalanine at 1 mmol/kg fat-free mass each (closed triangles). (b) Net-integrated AUC using the fasting values as baseline. Bars with different letters indicate that values are significantly different (*P* < 0.05).

The mean fasting glucagon concentration was 48 pg/mL (Figure 3(a)). After ingestion of glucose alone the plasma glucagon concentration decreased as expected and remained below the initial fasting value for the duration of the study. In contrast, ingestion of leucine + phenylalanine alone resulted in an increase in the plasma glucagon concentration that was maintained for the duration of the study. The decrease in plasma glucagon concentration observed with glucose ingestion alone was markedly attenuated when glucose was co-ingested with leucine + phenylalanine.

The glucagon area response (Figure 3(b)) was negative following ingestion of glucose alone. However, it was positive after ingestion of leucine + phenylalanine both with and without glucose. Indeed, the glucagon-lowering effect of glucose was totally negated when it was co-ingested with the two amino acids and was positive when compared to the water control. When leucine + phenylalanine was ingested with glucose, the glucagon area response increased by 165% compared to ingestion of glucose alone (*P* = 0.018). The glucagon area response after ingestion of leucine + phenylalanine alone was significantly greater than the water control (*P* = 0.014).

## 4. Discussion

In the present study, the insulin response to ingestion of leucine + phenylalanine with glucose was 77% greater than when the same amount of glucose was ingested alone. In our previous studies, we determined that ingestion of leucine alone with glucose, or phenylalanine alone with glucose, resulted in 66% and 7% greater insulin area responses, respectively, when compared to ingestion of the same amount of glucose alone [2, 3]. Thus, the increase in insulin area response with ingestion of leucine + phenylalanine with glucose was additive when compared to the amino acids ingested individually with glucose (leucine = 66%, phenylalanine = 7%, and leucine + phenylalanine = 77%). 

A similar pattern was seen when the two amino acids were ingested without glucose. The insulin area response to the combination of leucine + phenylalanine was 39% of that observed with glucose alone. In our previous studies, we found that ingestion of leucine or phenylalanine independently resulted in insulin area responses that were 10% and 19%, respectively, of the insulin area response to glucose alone [2, 3]. Thus, the insulin area response following ingestion of leucine + phenylalanine without glucose was additive and possibly even synergistic with respect to the individual amino acids when ingested without glucose (leucine = 10%, phenylalanine = 19%, and leucine + phenylalanine = 39%). 

Preliminary unpublished dose-response data from our laboratory indicate that the independent metabolic effect of these amino acids on insulin and glucose concentrations is likely to be near maximal using the dose of 1 mmol/kg fat-free body mass. If this is true, our data suggest that leucine and phenylalanine stimulate a rise in insulin by different (independent) mechanisms. This is consistent with other data in the literature regarding the mechanisms of action of leucine and phenylalanine.

When given intravenously in very large amounts (30 gm) leucine, arginine, lysine, phenylalanine, valine, and methionine, but not histidine, were reported to stimulate a rise in circulating insulin concentration [4]. Thus, these amino acids can directly stimulate insulin secretion at least when given in large amounts. However, a potential mechanism or mechanisms by which they raise the insulin concentration have not been determined to our knowledge, with the exception of leucine. 

There is a large literature regarding the mechanism and/or mechanisms by which leucine could stimulate insulin secretion both directly and indirectly. Most have centered on its effects on mitochondrial metabolism. A review of these data is beyond the scope of this paper but it is clear that leucine is an important regulator of insulin secretion and thus of glucose as well as amino acid metabolism [2, 4–6].

The mechanism by which glucose ingestion further strongly facilitates a rise in insulin concentration beyond that stimulated by leucine alone remains unknown. The facilitation of a rise in insulin concentration by glucose when ingested with other amino acids also remains mechanistically unknown.

The importance of glucose ingestion in facilitating a large increase in insulin by leucine is demonstrated by our previous observation that the leucine concentration remained grossly elevated for the duration of the study, but the insulin concentration decreased and returned to a fasting level in association with the decrease in glucose concentration [2]. 

In summary, in our previous study when leucine was ingested with glucose, the quantitative and dynamic characteristics of insulin secretion could be explained by an additive stimulation of insulin secretion by both glucose and leucine.

 Compared to leucine, much less is known about the mechanism by which phenylalanine reduced the glucose response when ingested with glucose. When given intravenously in a very large amount, (30 gm) phenylalanine was just as potent as intravenous leucine in stimulating a rise in insulin concentration [4]. However, in *in vitro* data using rat pancreas/islets, investigators reported that phenylalanine had no effect on insulin secretion [7–9]. To our knowledge, this has not been confirmed in human islets. Phenylalanine also did not stimulate GLP-1 secretion from mouse colonic cells *in vitro* [9]. 

Our previous data (Figure 2, insert) [1, 3] suggest that phenylalanine stimulation of insulin secretion is relatively more potent than leucine. However, in contrast to leucine, ingested glucose does not facilitate a significant potentiating effect on insulin secretion. The ingested phenylalanine did not affect the rise in glucose but dramatically accelerated the return of the glucose concentration to the fasting value. This suggests that phenylalanine is accelerating the removal rate of circulating glucose under these conditions and/or is profoundly inhibiting endogenous glucose production. If so, phenylalanine does so by an as yet to be determined mechanism. That is, it could have an independent glucose-lowering effect. Also theoretically, it could result in an increase in organ sensitivity to insulin. However, why the continued insulin elevation did not result in hypoglycemia suggests the development of a degree of insulin resistance. 

The delayed decrease cannot be explained by either the glucose or phenylalanine concentration. The phenylalanine concentration remained elevated and glucose had returned to the baseline [3]. Clearly, the mechanisms by which these amino acids affect glucose disposal are different, and for phenylalanine, they are complex and poorly understood.

In the present study, when the combination of leucine + phenylalanine was ingested with glucose, the glucose dynamics resembled a modification of the response to phenylalanine, with the glucose returning to the baseline 20 minutes before the insulin (Figure 1, insert). In addition, the decrease in glucose concentration occurred when the insulin concentration was still ~40% of its maximal value.

Since leucine and phenylalanine appear to lower blood glucose by different mechanisms, an additive effect on the glucose response was expected when they were given together. Why the decrease was not greater is unclear. It may at least in part, be due to a limit in the amount of reduction possible under any circumstances. In other words, the rate of glucose absorption may exceed the ability of the increased insulin to stimulate glucose removal and/or suppress endogenous glucose production. Alternatively, and more likely, it suggests that one amino acid is inhibiting the glucose-lowering effect of the other.

 It should be noted that the attenuation in glucose area response when the two amino acids were ingested with glucose occurred, while the glucagon concentration remained elevated. It also should be noted that the glucagon concentration was still significantly elevated at the end of the study in spite of a stable or lower glucose concentration (Figure 3). As we have pointed out in previous publications (see [3]), the role of glucagon in the regulation of plasma glucose, if any, remains unclear, particularly in the postprandial period. It certainly does not appear to play a role in the dynamics of glucose disposal and/or endogenous glucose production in the present study. 

## 5. Conclusion

 Despite an additive effect on insulin area response and the less than additive effect on glucagon area response that was observed with this amino acid pair, coingestion of leucine + phenylalanine with glucose did not produce an additional lowering of the glucose area response compared to ingestion of each amino acid independently. Current data suggest that leucine and phenylalanine increase insulin by different mechanisms. Phenylalanine also may have a glucose-lowering effect not directly mediated by a rise in insulin. Nevertheless, there is an unknown factor preventing them from interacting to further attenuate a rise in plasma glucose when glucose is ingested.
